# HIV-1 Low-Frequency Variants Identified in Antiretroviral-Naïve Subjects with Virologic Failure after 12 Months of Follow-Up in Panama

**DOI:** 10.3390/idr15040044

**Published:** 2023-08-01

**Authors:** Ambar Moreno, Claudia González, Jessica Góndola, Oris Chavarría, Alma Ortiz, Jorge Castillo, Juan Castillo Mewa, Juan Miguel Pascale, Alexander Augusto Martínez

**Affiliations:** 1 Department of Research in Genomics and Proteomics, Gorgas Memorial Institute for Health Studies, Panama City 0816-02593, Panama; ammoreno@gorgas.gob.pa (A.M.);; 2 Department of Microbiology and Immunology, University of Panama, Panama City 3366, Panama

**Keywords:** HIV-1, low-frequency variants, virologic failure, next-generation sequencing, drug resistance, Panama

## Abstract

Low-frequency mutations associated with drug resistance have been related to virologic failure in subjects with no history of pre-treatment and recent HIV diagnosis. In total, 78 antiretroviral treatment (ART)-naïve subjects with a recent HIV diagnosis were selected and followed by CD4+ T lymphocytes and viral load tests to detect virologic failure. We sequenced the basal samples retrospectively using next-generation sequencing (NGS), looking for low-frequency mutations that had not been detected before using the Sanger sequencing method (SSM) and describing the response to ART. Twenty-two subjects developed virologic failure (VF), and thirteen of them had at least one drug-resistance mutation associated with Reverse Transcriptase Inhibitors (RTI) and Protease Inhibitors (PIs) at frequency levels ≤ 1%, not detected previously in their basal genotyping test. No resistance mutations were observed to Integrase Strand Transfer Inhibitors (INSTIs). We identified a possible cause of VF in ART-naïve subjects with low-frequency mutations detected. To our knowledge, this is the first evaluation of pre-existing drug resistance for HIV-1 minority variants carried out on ART-naïve people living with HIV/AIDS (PLWHA) by analyzing the HIV-1 *pol* gene using NGS in the country.

## 1. Introduction

The presence of low-frequency drug resistance mutations is associated with HIV virologic failure (VF) in subjects without previous history of antiretroviral treatment (ART) [1]. This situation is commonly observed in those individuals starting ART regimes based on Non-Nucleoside Reverse Transcriptase Inhibitor (NNRTI), known as pre-treatment drug resistance (PDR) [2]. Next-generation sequencing (NGS) is a massive sequencing methodology that provides high sensibility and capability to identify changes in HIV quasispecies at frequencies as low as 1% [3]. However, the Sanger sequencing genotyping test does not detect low-frequency mutations present in <20% or less of the viral population, a widely used method for resistance testing [4]. Improvements in the clinical management of people living with HIV/AIDS (PLWHA) have been reported based on NGS detection of low-frequency mutations [5,6].

In Panama, the first study that evaluated the prevalence of transmitted drug resistance (TDR) was performed in 2011, showing 5–15% levels. In 2014 it was 9.2%, and by 2018 it increased to 15.2% [7,8,9]. Most of that resistance was associated with Efavirenz (EFV) and Nevirapine (NVP). In 1999, Panama began to use Disoproxil Fumarate (TDF), Lamivudine (3TC), and Efavirenz (EFV) as a first-line therapy scheme for adults [10]. Since 2016, the country began implementing a new “test and treat” strategy following the 90-90-90 WHO guidelines. However, an analysis of the baseline genotyping profile of low-frequency mutations that could be associated with resistance in the treatment of näive subjects has not been assessed in our country.

We evaluated the presence of pre-existing HIV-1 low-frequency mutations associated with antiretroviral drug resistance using NGS in 22 HIV-1-positive ART-naïve subjects who developed virologic failure and were initially susceptible to antiretroviral drugs.

## 2. Materials and Methods

### 2.1. Study Population

The Gorgas Memorial Institute for Health Studies (GMI) is the national reference laboratory for HIV monitoring tests in Panama. In 2016, subjects ≥ 18 years with a recent diagnosis of HIV viral infection (less than one year) that attended GMI for their first monitoring test were invited to participate in the study. The study was conducted in accordance with the Declaration of Helsinki. The protocol was approved by the Gorgas Memorial Institute Ethics Committee (Project N°1044/CBI/ICGES/2015). All subjects that gave written consent were subjected to baseline screening tests using commercial kits for CD4+ T-cell counts and HIV-1 viral load and an in-house Sanger sequencing methodology to determine the genotypic resistance profile. Participants completed a demographic form with data such as age, sex, residence, HIV diagnostic date, and a survey about HIV risk factors. All these subjects were diagnosed in 2016 and started the ARV scheme Disoproxil Fumarate (TDF), Lamivudine (3TC), and Efavirenz (EFV) according to the HIV-1 guidelines for the adult population in Panama.

**Figure 1:**
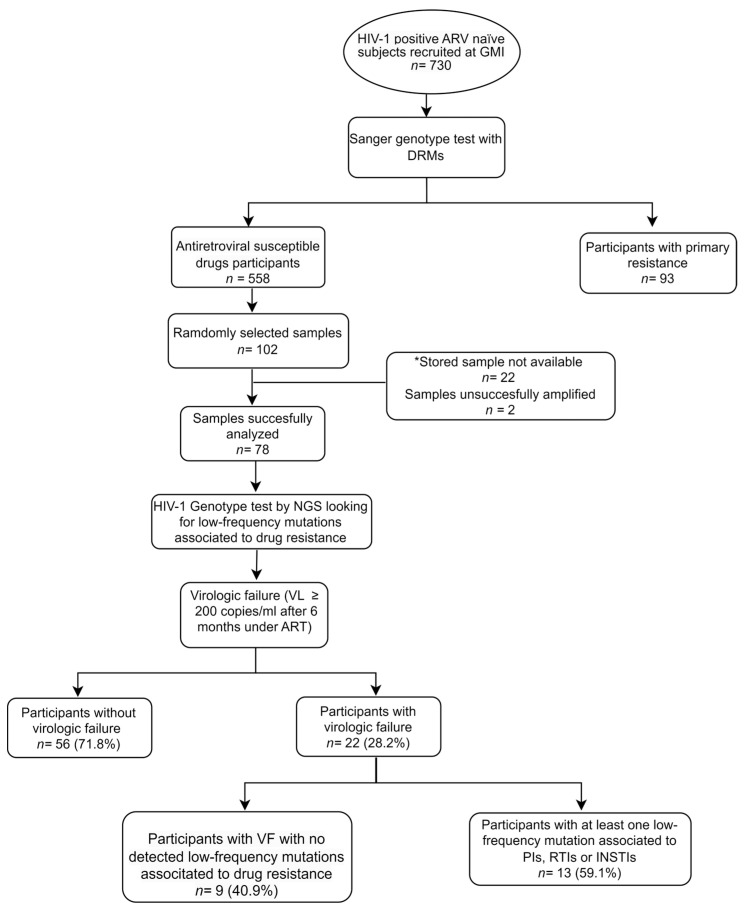
Flow chart showing subjects from selection to analysis.

Inclusion criteria for this study were established as follows: HIV-1 subjects over 18 years old, signed written consent, recent HIV-1 diagnosis (no more than one year), no history of previous ART, HIV-1 genotype classified as susceptible to antiretroviral drugs determined by Sanger genotyping test, and a first HIV-1 viral load greater than 1000 copies/mL. (For additional details about sample selection, see Figure 1).

Figure 1 Subjects were enrolled at their first monitoring test at GMI (Gorgas Memorial Institute) and had no history of previous ART. Participants classified in the group of ART-susceptible (no DRM detected using Sanger methodology) were selected to perform random sub-sample selection to seek low-frequency mutation using NGS methodology. Subjects who experienced virologic failure were analyzed for low-frequency mutation at levels ≤ 1%.

### 2.2. HIV-1 RNA Viral Extraction, pol Gene Amplification, and NGS Methods

RNA was obtained from plasma with EDTA using a QIAmp viral RNA mini kit (Qiagen, Germantown, TN, USA). A~2.8 kb fragment of HIV-1 *pol* gene amplification was obtained of protease (PR, codons 1–99), reverse transcriptase (RT, codons 1–560), and integrase (1–288), by performing an RT-PCR protocol without adding EzDnase as previously described [11]. Samples not successfully amplified with this protocol were amplified with a second in-house methodology, previously standardized in the GMI Genomics and Proteomics Department (See Table S1) [12]. We obtained a 1.2 kb fragment of the HIV-1 *pol* gene, including protease (PR codons 1–99) and partial reverse transcriptase (RT, codons 1–260). Pair-end libraries were prepared using a 96-sample Nextera^®^ XT DNA Library Preparation Kit (Illumina Inc., Hayward, CA, USA) following the manufacturer’s instructions and sequenced 500 cycles V2 in the Miseq system (Illumina Inc.). Information about bioinformatics, statistical analysis, and PCR assay is described in the supplementary material.

## 3. Results

### 3.1. Follow-Up of Subjects with Virologic Failure

**Figure 2:**
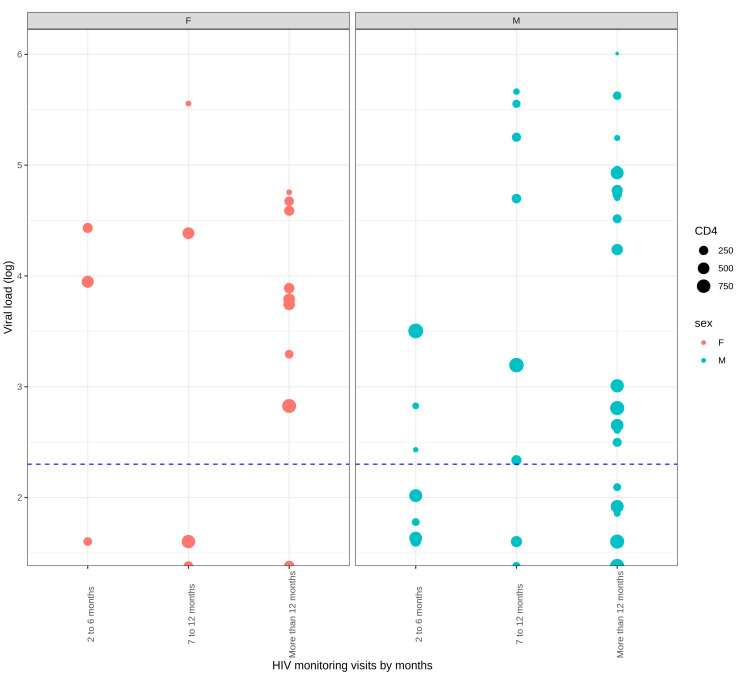
Distribution of HIV-1 viral load, CD4+ T lymphocytes count, and sex in subjects with virologic failure after a follow-up.

Of the 102 subjects evaluated, 78 (76.4%) were successfully amplified. Of these, twenty-two (22, 28.2%) were considered to have VF. HIV-1 viral load results and CD4+ T count were compared among gender through time in this group of subjects (Figure 2). Socio-demographic features between subjects with and without virologic failure were compared (Table 1). The sociodemographic features of those subjects who developed VF with and without low-frequency mutations were also compared (Table S2).

Figure 2. Samples were grouped by months according to HIV-1 monitoring visits and divided into males and females on the figure. The size of the dot represents the CD4+ T lymphocyte count, and the dashed line in blue shows 200 copies/mL. Of 22 subjects with VF, 16 were men (72.7%), and 6 were women (27.3%). No marked difference was shown in CD4+ T count between female and male subjects, showing values < 500 copies/mL throughout follow-up. Comparing HIV-1 viral load levels versus gender, the proportion of females failing to reach viral suppression was more significant than men, with a *p*-value of 0.006.

Table 1. In this study, the Republic of Panama was divided into four regions: metropolitan (Comprised Panama City and Chorrera); west Panama (Chiriquí, Bocas del Toro, and Ngäbe Buglé); central Panama (comprised Veraguas, Los Santos, Herrera, and Coclé); and east Panama (Comprised Darien, Guna Yala, and Colon). MSM: men who had sex with men.

### 3.2. Detection and Identification of Low-Frequency Mutations in Subjects with Virologic Failure

After analyzing the amino acid mutation reports of 22 subjects identified with VF, we identified minority DRM in a frequency ≤ 1% of viral quasispecies (Table 2). Of them, 11 (50%) subjects were identified with at least one mutation associated with nucleoside reverse transcriptase inhibitor (NRTI), and the most common mutation detected was D67E. Seven subjects had at least one mutation for non-nucleoside reverse transcriptase inhibitors (NNRTI), and the most common was P225H. TAMs were not identified in this study. Six subjects were identified with at least one major DRM to PIs; the most common was M46I. Two subjects were identified with low-frequency mutations to PIs, RTIs, and INSTIs. Finally, for INSTI analysis, two subjects with VF were identified with accessories mutations as L74M, Q95K, and V151I in low-frequency. No major DRMs were identified for integrase. All low-frequency mutations were detected with a deep greater than 17,000 X. We observed some mutations in higher frequency levels; however, no significant changes were detected against the first-line antiretroviral drugs used in Panama.

Table 2. Detected mutations in the *pol* gene on subjects with virologic failure in a frequency ≤ 1% of viral quasispecies. The table shows a prediction of the ART score of the Stanford University Database based on the amino acid mutation report. The ARV drugs were selected based on the first- and second-line schemes prescribed for adult HIV-1 ART-naïve subjects in Panama. 

## 4. Discussion

In this study, after following 78 PLWHA for 12 months, 22 subjects developed virologic failure (viral load > 200 copies/mL after six months of treatment). Of these 22 subjects, 13 had at least one low-frequency resistance mutation to NRTIs (D67E, T69D, L74I, V75EI, and F77L), to NNRTIs (G190E), or accessory mutations such as V108I, V106I, P225H, and N348I. Together, these resistance mutations cause high or intermediate resistance to Efavirenz (EFV), potential low resistance or low resistance to Tenofovir (TDF), and potential low-level resistance to Emtricitabine (FTC). All these drugs were included in the primary prescription scheme for ART-naïve subjects in Panama (ATRIPLA (FTC+ TDF+ EFV)) from 1999 to 2019 and continue to be the preferred prescription for the adult population infected with tuberculosis and for the inmate population [10]. The introduction of the TLD scheme (Tenofovir (TDF) + Lamivudine (3TC) + Dolutegravir (DTG)) in 2019 warrants the effectiveness of ART and decreases the probability of developing and transmitting drug-resistance mutations, as has been previously described [13,14].

P225H mutation was identified in three subjects and has been previously reported at low levels in subjects with virologic failure [15]. It is classified as a PDR and EFV-selected mutation that usually appears in combination with K103N; however, this mutation causes intermediate resistance to EFV and NVP, both ART drugs of the first-line scheme.

Not surprisingly, we observed very few mutations to PIs, an expected result due to the high genetic barrier of this class of drugs. However, we identified the M46I as the most common mutation to PIs, also reported at a low frequency in ART-naïve individuals in Mexico, which is associated with reduced susceptibility to ATV and LPV [16]. The N88S mutation, conferring high-level resistance to ATV, was detected in one subject. These primary mutations decreased the effectiveness of the second-line schemes used in Panama, consisting of using two NRTI + PIs, such as LPV/RTV/ATV. According to Panama’s alternative ARV regimen, second-line schemes were applied to subjects with an initial treatment failure to ATRIPLA or with treatment failure to AZT/3TC + EFV.

A few accessory mutations were identified in the integrase region, and no major level resistance was observed, consistent with other studies [17,18,19]. The L74M polymorphic mutation was commonly observed with a frequency of 0.5–20%, decreasing the effectiveness of the first- and second-generation INSTI drugs, as was previously reported in Brazil in a study of PDR on ART-naïve subjects [20].

The association between virologic failure and low-frequency mutations < 20% has been controversial, since some studies reported no evidence of association while others found evidence of effects on the susceptibility to ARV treatment.

Our preliminary results suggest that low-frequency mutations could impair the efficacy of first-line schemes, essentially against individuals under RTI drugs and who had no history of ARV treatment, and could be the unknown reason for virologic failure, as reported previously [15,21,22,23]. An observational study evaluated the impact of PDR mutations in low- and high-frequency levels on HIV-1-naïve individuals under the EFV ARV scheme for two years and observed an increased risk of virologic failure within a year after starting antiretroviral treatment [24]. In contrast, a study conducted on ARV-naïve subjects from Botswana reported a high prevalence of low-frequency DRM in this population; however, no impact was observed at the lower threshold of 1% of the viral quasispecies [25]. Other studies describe no association between pre-existing low-frequency DRMs and virologic failure [26,27,28].

This study had some limitations that should be considered when interpreting the results. First, the small number of participants with VF, including only 22 subjects, could limit our final analysis. Second, sequencing the sample at the virologic failure point was not possible because the failure to achieve virological suppression is related to treatment adherence; however, information regarding the ARV of these subjects was not available. Finally, we could not conduct an additional survey of the studied subjects to discover their concerns or challenges with receiving ARV treatment since factors such as adverse effects and shortages are very important when considering a failure in its effectiveness.

## 5. Conclusions

To our knowledge, this is the first evaluation carried out in ART-naïve subjects in adult PLWHA analyzing the entire HIV-1 *pol* gene in our country. We detected low-frequency mutations associated with resistance to RTIs and PIs and accessories mutations to INSTIs not detected in the basal genotyping test. No level of resistance was observed to INSTIs.

We identified a possible cause of the VF in 22 subjects analyzed in this study over six months under ART, who did not sustain viral suppression under 200 copies/mL. Further studies are recommended to evaluate the evolution of the low-frequency mutations identified in this group of subjects by analyzing the virologic point sample with a high-sensibility sequencing methodology such as NGS.

These findings will guide clinical care to create strategies to provide individualized, efficient, and more specific ARV treatment and follow-up care in subjects unable to achieve sustained viral suppression. Additionally, we demonstrated the impact of detecting DRMs at low frequencies to make clinical decisions based on NGS sequencing methodologies with high specificity and sensibility.

## Figures and Tables

**Table 1:** Comparison of sociodemographic features between subjects with and without virologic failure.

	Participants without VF (*n* = 56)	Participants with VF (*n* = 22)
Features		
Sex, n (%)		
Male	45, 80.4	16, 72.7
Female	11, 19.6	6, 27.2
Average age (years), median age (min–max)		
Men	30 (18–53)	29 (20–53)
Women	31 (18–38)	27 (19–45)
Baseline CD4+ T lymphocyte count		
Median	340 cells/µL	301 cells/µL
Min–max	54–1265 cells/µL	10–1196 cells/µL
Viral load		
Median	log_10_ 4.8 copies/mL	log_10_ 4.8 copies/µL
Min–max	log_10_ 3.5–6.2 copies/mL	log_10_ 3.1–6.2 copies/mL
Nationality, n (%)		
Panamanian	45 (80.4)	22 (100)
Colombian	6 (10.7)	-
Venezuelan	3 (5.4)	-
Nicaraguan	2 (3.6)	-
Residence, n (%)		
Panama City	49 (87.5)	19 (86.4)
West Panama	5 (8.9)	-
Central Panama	2 (3.6)	1 (4.5)
East Panama	-	2 (9.1)
Risk group, n (%)		
Heterosexual	26 (46.4)	12 (54.5)
MSM	27 (48.2)	9 (41.0)
Bisexual	3 (5.4)	1 (4.5)

**Table 2:** Low-frequency mutations identified using NGS in subjects with VF associated with resistance to PIs, RTIs, INSTIs, and their prediction according to the Stanford HIV database score.

Mutations Detected in *pol* Gen						Baseline Genotypic Level of Resistance According to Antiretroviral Drug Based on NGS Data												
Accession No.	SSM	NGS Method				Protease Inhibitors			Reverse Transcriptase Inhibitors							Integrase Inhibitors		
		PR	RT		IN	ATV	DRV	LPV	ABC	3TC	FTC	TDF	AZT	EFV	NVP	BIC	DTG	RAL
OP726028	None	**G48V, I54T**	D67E	None	None	IR	S	LLR	S	S	S	S	PLLR	S	S	S	S	S
OP726030	None	None	D67E	P225H	None	S	S	S	S	S	S	S	PLLR	IR	IR	S	S	S
OP726033	None	None	D67E	None	None	S	S	S	S	S	S	S	PLLR	S	S	S	S	S
OP726045	None	**M46I**	F77L	None	V151I	PLLR	S	PLLR	S	S	S	S	PLLR	S	S	S	S	S
OP726046	None	**M46I**	None	E138K	None	PLLR	S	PLLR	S	S	S	S	S	S	PLLR	S	S	S
OP726048	None	None	D67E	None	None	S	S	S	S	S	S	S	PLLR	S	S	S	S	S
OP726009	None	None	None	**G190E**	None	S	S	S	S	S	S	S	S	HLR	HLR	S	S	S
OP726051	None	None	D67EN, **L74I**	V106I	None	S	S	S	IR	S	S	PLLR	LLR	S	PLLR	S	S	S
OP726059	None	**M46I/L**	D67E, L74IV, V75M, F77L	P225H	None	PLLR	S	PLLR	IR	PLLR	PLLR	LLR	IR	IR	IR	S	S	S
OP726062	None	**N88S**	**L74I**, F77L,	P225H	L74M, Q95K	HLR	S	S	IR	S	S	LLR	LLR	IR	IR	S	S	PLLR
OP726068	None	**M46L**	D67E	None	None	PLLR	S	PLLR	S	S	S	S	PLLR	S	S	S	S	S
OP726076	None	None	D67E, **L74I**, V75I, F77L	V108I, N348H,	None	S	S	S	IR	PLLR	PLLR	LLR	IR	PLLR	LLR	S	S	S
OP726080	None	None	D67E	None	None	S	S	S	S	S	S	S	PLLR	S	S	S	S	S

Bold: major drug resistance mutation (DRM), underlined: transmitted drug resistance mutation (TDR). Abbreviations—SSM: Sanger sequencing method, NGS: next-generation sequencing, PR: protease, RT: reverse transcriptase, IN: integrase, ATV: Atazanavir, DRV: Darunavir, LPV: Lopinavir, ABC: Abacavir, 3TC: Lamivudine, FTC: Emtricitabine, TDF: Tenofovir, EFV: Efavirenz, NVP: Nevirapine, BIC: Bictegravir, DTG: Dolutegravir, RAL: Raltegravir, S: susceptible, PLLR: potential low-level resistance, LLR: low-level resistance, IR: intermediate resistance, HLR: high-level resistance. Bold: major mutation. Underlined: PDR mutation. Gray columns: ARV therapy started for all subjects according to Panama guidelines.

## Data Availability

The data supporting this study’s findings are available from the corresponding author upon reasonable request.

## Supplementary Materials

The following supporting information can be downloaded at: https://www.mdpi.com/article/10.3390/idr15040044/s1, Table S1: PCR primers for HIV-1 *pol* gene amplification (PR, RT, and IN). Table S2: Comparison of sociodemographic features between subjects with and without low-frequency mutation associated with drug resistance that developed virologic failure. References [11,29,30,31] are cited in the supplementary materials.
