# Supplementary material for: HIV-1 Low-Frequency Variants Identified in Antiretroviral-Naïve Subjects with Virologic Failure after 12 Months of Follow-Up in Panama
Source: Infect Dis Rep. 2023 Aug 1;15(4):436–44. doi: 10.3390/idr15040044 (PMC10454674; doi:10.3390/idr15040044)
Supplement: Supplementary file 1 [file idr-15-00044-s001.zip › idr-2406250-supplementary.docx]

**Materials and methods**

**Description of the study population**

A total of 730 participants were recruited; HIV-1 mutations associated with drug resistance were not identified in 558 (76.4%) subjects and were classified as ART drug-susceptible, 93 (12.6%) were classified as ART drug-resistance after detecting mutations associated with drug resistance with the Sanger genotyping test, 79 (7.1%) were not considered for a genotyping test due to low viral load (less than 2000 copies/ml) and 27 (3.69%) were not successfully amplified. For this study, we randomly selected a sub-sample of 102 subjects from the ART drug-susceptible group by using the online tool Win Epi 1.0 [1]. The samples randomly selected for this study were storage samples with no minority mutations detected on their first genotyping test.

**PCR assay**

- HIV-1 *pol* gene amplification, including protease (PR) and reverse transcriptase (RT)

The RT-PCR utilized 8 µl of RNA extracted from HIV-1 plasma to amplify HIV-1 *pol* (PR and RT), 3798 primer at 10 pmol, nuclease-free water, and dNTPs were first incubated for 5 minutes at 65°C to unfold the RNA, following by incubation on ice. After incubation, nuclease-free water, Super Script IV RT enzyme, Super Script buffer IV, dithiothreitol (DTT), and RNase inhibitor were added to complete a final volume of 20 µl. The thermocycling conditions for the RT-PCR were; one cycle at 50°C for 60 minutes, 85°C for 5 minutes, and ended at 4°C.

For the first PCR amplification, 5 µl of cDNA, nuclease-free water, Platinum Taq High Fidelity enzyme, JA269 and JA272 primers at 10µM, 10X PCR buffer, MgSO4 at 50nM, and dNTPs at 10nM were added to complete a final volume of 50µl. For nested PCR, the primers used were JA270 and JA271 (See Table S1 for further details).

The thermocycling conditions for the first PCR started at 94°C for 2 minutes for inactivation of the reverse transcriptase, 30 cycles at 94°C for 20 seconds, 50°C for 20 seconds for the annealing step, and 72°C for 1 minute 30 seconds for final elongation. Finally, 5 minutes at 72°C and storage at 4°C. The thermocycling conditions for nested PCR were the same as PCR, with one change in the number of cycles for hybridization and elongation for 40 cycles.

- HIV-1 *pol* amplification, including protease (PR), reverse transcriptase (RT), and integrase (IN)

The RT-PCR utilized 10 µl of RNA extracted from HIV-1 plasma to amplify HIV-1 *pol* (PR, RT, and IN), were firstly incubated for 5 minutes at 65°C to unfold the RNA, followed by incubation on ice. After the incubation, nuclease-free water, Super Script IV RT mix, 2X Platinum SuperFi PCR master mix, Super Script buffer IV, and primers 5575 and 1810 at 20 pmol were added for a final volume of 50 µl. The thermocycling conditions for the touchdown RT- PCR started at 65°C for 5 minutes to unfold the RNA, 50°C for 10 minutes to inactivate the reverse transcriptase, 98°C for 2 minutes, 10 cycles at 98°C for 10 seconds, gradient step of 62-52 °C for 10 seconds, and 72°C for 2 minutes), 30 cycles of 98°C for 10 seconds, 52 °C for 10 seconds and 72°C for 2 minutes), 72 ° for 5 minutes for final elongation and storage at 4°C.

For the nested touchdown PCR, 5 µl of the primary reaction product, nuclease-free water, dNTPs at 10mM, MgSO4 at 50nM, Platinum Taq HiFi enzyme, 10X PCR buffer, 2006 and 5264 primers at 20 pmol were used to obtain a final volume of 50 µl. The thermal cycler conditions for nested touchdown PCR started at 94°C for 2 minutes for denaturation of DNA and activation of Taq HiFi polymerase, initial amplification of 10 cycles at 94°C for 30 seconds, gradient cycling at 63-53°C for 30 seconds, and elongation at 72 °C for 3:30 minutes. Final amplification started with 30 cycles of denaturation at 94°C for 30 seconds, an annealing step at 53°C for 30 seconds, and elongation at 72°C for 3:30 minutes. Finally, 5 minutes at 72°C and storage at 4°C.

Table S1. PCR primers for HIV-1 *pol* gene amplification (PR, RT, and IN).

| **Primers** | **Gene** | **Sequence** | **Position** | **Amplicon length** | **Reference** |
| --- | --- | --- | --- | --- | --- |
| **RT** |  |  |  |  |  |
| 3798 (R) | *pol** | 5’CAAACTCCCACTCAGGAATCCA3’ | 3780-3801 |  | [2] |
| **PCR 1** |  |  |  |  |  |
| 269 (F) | *pol** | 5’AGGAAGGACACCARATGAARGA3’ | 2043-2064 | 1323 pb | [2] |
| 272 (R) |  | 5’GGATAAATCTGACTTGCCCART3’ | 3345-3366 |  |  |
| **Nested PCR** |  |  |  |  |  |
| 270 (F) | *pol** | 5’GCTTCCCTCARATCACTCTT3’ | 2249-2268 | 1086 pb | [2] |
| 271 (R) |  | 5’CCACTAAYTTCTGTATRTCATTGAC3’ | 3311-3335 |  |  |
| **RT PCR** | | | | | |
| 1810 (F) | *pol*** | 5’GCTACAYTAGAAGAAATGATGACAGCATG3’ | 1810-1838 | 3766 pb | [3] |
| 5575 (R) |  | 5’TCTGGGGCTTCCATCTATC3’ | 5554-5575 |  |  |
| **PCR** | | | | | |
| 2006 (F) | *pol*** | 5’GGGCCCCTAGGAAAAAGGG3’ | 2006-2024 | 3259 pb | [3] |
| 5264 (R) |  | 5’CCTGTATGCAGACCCCAATATGTT3’ | 5241-5264 |  |  |

*pol** includes protease and reverse transcriptase. *pol**:* include protease, reverse transcriptase, and integrase.

**Bioinformatics analysis**

Raw data FASTAQ files were analyzed using a pipeline developed for HIV-1 associated resistance based on HyDRA quasitools web version 1.7.0 and Stanford University HIV Drug Resistance Database scores (<http://hivdb.stanford.edu>). After that, we obtained the amino acid mutation report (aavfiles format) and their frequency for each sample to analyze with the HIVdb program genotyping resistance interpretation algorithm from the Stanford University HIV Drug Resistance Database to predict the levels of susceptibility. We classified them according to the codon frequency changes: frequency ≥ 5%, frequency between 1-5 %, and frequency ≤ 1% using the RStudio version 4.1.0. We considered a variant in low-frequency mutations with coverage greater than 0.03% (to filter Miseq error rate of 0.03%) and a deep greater than 17 000 X. Each codon change was also classified as a Drug Resistance or Surveillance Category.

**Statistics analysis**

Statistical analyses were performed using RStudio version 4.1.0; a proportion test was used to evaluate the association between a major level of viral suppression and gender [4]. *p-*value < 0.05 was considered statistically significant.

Table S2. Comparison of sociodemographic features between subjects with and without low-frequency mutation associated with drug resistance that developed virologic failure.

|  | Participants without low-frequency mutations (n=9) | Participants with low-frequency mutations (*n*=13) |
| --- | --- | --- |
| Features |  |  |
| Sex gender, n (%) |  |  |
| Male | 7 (77.8%) | 9 (69.2%) |
| Female | 2 (22.2%) | 4 (30.8%) |
| Average age (years), median age (min-max) |  |  |
| Men | 25 (20-38) | 32 (27-53) |
| Women | 20 (19-21) | 30 (19-45) |
| Baseline CD4+ T lymphocyte count |  |  |
| Median | 200 cells/µl | 340 cells/µl |
| Min-max | 62-1196 cells/µl | 10- 730 cells/µl |
| Viral load |  |  |
| Median | log_10_ 4.5 copies/ml | log_10_ 4.7 copies/µl |
| Min-max | log _10_ 3.1- 5.9 copies/ml | log _10_ 4.4- 6.2 copies/ml |
| Nationality n (%) |  |  |
| Panamanian | 9 (100%) | 13 (100%) |
| Residence |  |  |
| Panama City | 8 (88.9%) | 11 (84.6%) |
| Central Panama | 1 (11.1%) | - |
| East Panama | - | 2 (15.4%) |
| Risk group |  |  |
| Heterosexual | 4 (44.4%) | 8 (61.5%) |
| MSM | 5 (55.6%) | 4 (30.8%) |
| Bisexual | - | 1 (7.7%) |

References

1. Ignacio de Blas. Working in Epidemiology [Internet]. Universidad de Zaragoza. 2006 [cited 2022 Jun 8]. Available from: http://www.winepi.net/

2. Murillo W, de Rivera IL, Parham L, Jovel E, Palou E, Karlsson AC, et al. Prevalence of drug resistance and importance of viral load measurements in Honduran HIV-infected patients failing antiretroviral treatment. HIV Med. 2010;11(2):95–103.

3. Chrysostomou AC, Topcu C, Stylianou DC, Hezka J, Kostrikis LG. Development of a new comprehensive HIV-1 genotypic drug resistance assay for all commercially available reverse transcriptase, protease, and integrase inhibitors in patients infected with group M HIV-1 strains. Infect Genet Evol. 2020;81(December 2019):104243.

4. Newcombe RG. Two-sided confidence intervals for the single proportion: comparison of seven methods. Stat Med. 1998;24(21):3383–4.
